# Correction: Effects of hazard types on drivers' risk rating and hazard response in a video-based hazard perception task

**DOI:** 10.1371/journal.pone.0215744

**Published:** 2019-04-16

**Authors:** The PLOS ONE Staff

## Notice of Republication

This article was republished on April 9, 2019 to correct for significant typographical errors introduced during the typesetting process. The publisher apologizes for the errors. Please download this article again to view the correct version. The originally published, uncorrected article and the republished, corrected article are provided here for reference.

## Supporting information

S1 File Originally published, uncorrected article. (PDF)

S2 File Republished, corrected article. (PDF)
