# Supplementary material for: Correction: Effects of hazard types on drivers' risk rating and hazard response in a video-based hazard perception task
Source: PLoS One. 2019 Apr 16;14(4):e0215744. doi: 10.1371/journal.pone.0215744 (PMC6467391; doi:10.1371/journal.pone.0215744)
Supplement: S2 File — (PDF) [file pone.0215744.s002.pdf]

PLOS ONE

RESEARCH ARTICLE

Effects of hazard types on drivers' risk rating and hazard response in a video-based hazard perception task

Long Sun*, Lingsen Hua

School of Psychology, Liaoning Normal University, Dalian, Liaoning, P. R. China

* sunlong510@yeah.net

Abstract

Hazard perception is crucial for identifying potential hazards on the road, and how quick drivers can respond to the hazard partially relies on their risk rating of the hazard after they detect it. Although many studies have attempted to reveal the relationship between drivers' response latencies and their risk ratings, this relationship has not been extensively explored under different hazard types. The present study addresses this issue using a video-based hazard perception task. Forty novice drivers and 35 experienced drivers were recruited and 26 video clips containing either an overt hazard (continuous visibility) or a covert hazard (interrupted visibility) were shown to participants. Participants were asked to finish the hazard perception task first and then rated the risk level of the hazard when each video clip was replayed. Participants' confidence in their answers for risk ratings was also determined. Results showed that experienced drivers responded to overt and covert hazards faster than did novice drivers. A negative and significant correlation was found between drivers' risk ratings of covert hazards and their response latencies. Such a relationship was not found for overt hazards. More importantly, drivers rated the risk level of covert hazards higher than that of overt hazards, and higher risk ratings of covert hazards resulted in faster responses to these hazards. The findings of the present study indicate that hazard types not only influence drivers' risk ratings and response latencies but also determine their relationships.

Check for updates

OPEN ACCESS

Citation: Sun L, Hua L (2019) Effects of hazard types on drivers' risk rating and hazard response in a video-based hazard perception task. PLoS ONE 14(3): e0214226. https://doi.org/10.1371/journal.pone.0214226

Editor: Amanda N. Stephens, Monash University, AUSTRALIA

Received: October 2, 2018

Accepted: March 10, 2019

Published: March 21, 2019

Copyright: © 2019 Sun, Hua. This is an open access article distributed under the terms of the Creative Commons Attribution License, which permits unrestricted use, distribution, and reproduction in any medium, provided the original author and source are credited.

Data Availability Statement: The data are available from: 10.6084/m9.figshare.7519679.

Funding: This study was supported by the Doctoral Science Foundation of Liaoning Normal University (BS2018L011).

Competing interests: The authors have declared that no competing interests exist.

Introduction

Hazard perception is the ability to identify potentially hazardous situations on the road [1]. Hazard perception is widely recognized as a multi-component ability that involves visual detection, appraisal and classification [1,2]. When facing a specific hazardous situation, drivers must first visually detect the potential hazard and then evaluate the risk level of the hazard (possibility of causing a collision) based on their experience before an evasive action is needed. Despite the importance of visual detection of hazards, many studies have reported that the visual detection time of young novice drivers was not significantly different from that of experienced drivers or older experienced drivers in video-based hazard perception tasks [3,4]. However, studies have demonstrated that the time from novice drivers visually detected a

hazard to the moment that they made a response was significantly slower than that of experienced drivers [5,6]. Also, previous empirical evidence further suggested that after drivers visually detected a hazard,the subjective rating of the hazard played a larger part in determining their response time [7,8]. For instance, drivers' higher risk rating score of the hazards in a picture-based hazard perception test lead to faster responses [9]. Therefore, how quick drivers can respond to a hazard relies on their visual detection or/and risk rating of the hazard after they detect it.

Typically, hazard perception is measured using the reaction time paradigm. Participants are instructed to respond quickly to the hazards in video clips, shot from the drivers' perspective, when they detect them. Many studies have provided evidence supporting the notion that experienced drivers outperformed novice drivers in video-based hazard perception tasks [1,5,10], though with some exceptions [11–13].

However, based on the reaction time paradigm, drivers' response latencies to the hazards are unavoidably affected by their subjective risk thresholds [1,10]. A signal detection study found that untrained novice drivers responded to the hazards in the video clips significantly slower than trained novice drivers and experienced drivers. The differences in response latencies derived from their response bias, with untrained novice drivers were significantly more conservative than the other two driver groups. The results showed that untrained novice drivers had a higher risk threshold before they are willing to classify a situation as hazardous [10]. This was supported by a recent study showing that young drivers who had a higher risk threshold have longer response latencies than drivers who had a lower risk threshold [14]. Although many studies have attempted to assess drivers' subjective risk thresholds by examining their risk ratings of the hazards present in video clips or pictures [9,10,15,16], mixed results have been reported. Some studies have found that novice drivers rated the risk level of hazards significantly lower than experienced drivers [9,16], while others found no differences between novice drivers and experienced drivers [10,12].

This inconsistency in response latencies and risk ratings may be in part due to the hazard type used in previous studies [17]. Although many studies have found that the responses to some hazards are more sensitive to driving experience than other hazards [2,18], the effects of hazard types on drivers' subjective risk ratings have not been thoroughly explored. Thus, it is of great importance to identify the effects of hazard types on drivers' response latencies and their risk ratings of the hazards.

The main purpose of the present study was to examine the effects of hazard types on drivers' risk ratings and their response latencies by manipulating the visibility of hazards in a video-based hazard perception task. The second purpose was to reveal whether drivers' risk ratings corresponded to their hazard response latencies and whether the relationship between the two varied with hazard types. It was predicted that hazards with interrupted visibility will delay drivers' response latencies and lower their subjective risk ratings.

Methods

Participants

The study was approved by the Logistics Department for Civilian Ethics Committee of Liaoning Normal University. Seventy-five drivers who participated in the experiment were provided with and signed an informed consent form. Participants were recruited through a traffic video channel of Dalian. Participants were divided into two driver groups according to their driving experience since they obtained a valid driving license.

- Novice driver group: 40 drivers (18 males), whose age ranged from 19 to 27 years old with a mean age of 23.35 years (SD = 2.07), had an average of 0.76 years of driving experience (SD = 0.43).
- Experienced driver group: 35 drivers (18 males), whose age ranged from 30 to 55 years old with a mean age of 39.63 years (SD = 6.54), had an average of 12.83 years of driving experience (SD = 6.44).

The two driver groups were significantly different in their age (t (73) = -14.92, $p < 0.01$) and driving experience (t (73) = -11.83, $p < 0.01$); their gender ratio was not significantly different ($\chi^2 = 0.31$, $p > 0.05$). All participants had normal or corrected- to-normal vision.

Materials

A video-based hazard perception task developed by the first author was used [19]. The task contained 26 video clips (see Table 1). All video clips were filmed from a driver's perspective around the Dalian urban area under fine weather. Each video clip showed a traffic situation where a potential hazard was developing slowly as the camera car approached. The task has good discriminant validity in Chinese drivers [19].

The hazards in the clips were split into two types according to whether the visibility of the hazards was continuous or interrupted during their materialization [20]. Specifically, overt hazards were fully visible in the process of materialization in front of the camera car. Covert

Table 1. Information regardingthe hazards in the video clips.

Video clip	Hazard trigger	Clip Length(s)	Visibility	Brief description
1	Car	15	Continuous	A car in front signalled to turn right
2	Car	16	Continuous	A car in the next lane signalled to merge
3	Car	13	Continuous	A car in front slowed down
4	Car	14	Continuous	A car from the opposite direction crossed the path from the left
5	Car	15	Continuous	A car from the opposite direction crossed the path from the right
6	Car	12	Continuous	Head-on
7	Car	16	Interrupted	A van from the opposite direction crossed the path from the left and blocked a car behind, and the light was turning green
8	Car	15	Interrupted	A car from the side road merged into the main road but was blocked by trees
9	Car	13	Interrupted	In the opposite lane, a car ahead flashed to turn right but was blocked by the vehicles in front
10	Car	16	Interrupted	A car stuck in a jam ahead signalled to emerge into the next driving lane but was blocked by a truck
11	Car	14	Interrupted	A car broke down at the entrance of aside road and blocked another car that emerged onto the main road
12	Car	15	Interrupted	A sanitation car parked on the roadside but was blocked by the vehicle in front
13	Pedestrian	10	Continuous	A pedestrian crossed the road from the same side
14	Pedestrian	12	Continuous	A child ran into the driving lane
15	Pedestrian	13	Continuous	A pedestrian who stood on the safety island crossed the road in front
16	Pedestrian	10	Interrupted	A pedestrian crossed the road from the opposite side but was blocked by the vehicles in the other lanes
17	Pedestrian	14	Interrupted	A pedestrian crossed the road in front of a stopped bus
18	Pedestrian	12	Interrupted	A bus stopped at a stop, and a pedestrian entered the driving lane in front of it
19	Cyclist	14	Interrupted	A cyclist entered the road from a side road but was blocked by a van in front
20	Cyclist	13	Continuous	A cyclist rode in front rode along the road
21	Motorcyclist	10	Continuous	A motorcycle entered into the driving lane from a parking lot
22	Motorcyclist	10	Interrupted	A motorcycle from the opposite side crossed the road but was blocked by a van on the same side
23	Road work	12	Continuous	Road work was taking place ahead
24	Obstacle	12	Interrupted	Ahead of a long curve, an obstacle was not seen until the car approached

https://doi.org/10.1371/journal.pone.0214226.t001

hazards were partially or completely blocked at the very beginning of their materialization. In this study, twelve video clips contained overt hazards, and 12 video clips contained covert hazards. There are twenty-four hazards in the test. The other two video clips were used as practice clips.

Road types in the video clips were counter-balanced under each hazard type to minimize the effect of local familiarity. The length of the video clips ranged from 10 to 16 seconds, and differences in the mean length foreach hazard type were not significant, $t\ (22) = 0.62$, $p > 0.05$. The onset time and location of the hazards differed from one clip to another. A *hazard window* was defined for each hazard. The window began at the earliest point in time when the hazard was detectable and ended at the point when an avoidance response by the driver would fail to prevent a collision [4,13]. The beginning and ending points of the window were defined by three expert drivers (2 males and 1 female) with a high degree of agreement. The length of the hazard window ranged from 2 to 4.5 seconds. The mean length of the hazard window for each hazard type was not significant, t (22) = -0.75, $p > 0.05$.

Experimental design

A 2×2 mixed design was employed. The between-groups factor was driving experience. The within-groups factor was hazard type. The dependent variables were response rate, response latency, and risk rating score.

Procedure

Participants first finished a demographic questionnaire and watched two practice clips. They were instructed to click the left mouse button quickly when they detected a potential hazard that may lead to a collision with the camera car. Then, 24 video clips were randomly assigned to each participant on a 24-inch monitor at a resolution of 1280×720. In this study, custom software developed by the author's team was used to record participants' response behaviours. Finally, after finishing the hazard perception task, as in previous studies [9,10,15,16], participants rated the risk level of the hazard present in each video clip on a 5-point Likert scale ranging from *the risk can be ignored* (1) to *the risk was unavoidable* (5). Additionally, participants rated their confidence in their answers for risk ratings on a 5-point Likert scale ranging from *not confident at all* (1) to *very confident* (5). Each video clip was replayed once to assist participants in their risk rating. The experiment lasted approximately 25 minutes. Each participant received 50 yuan RMB after he/she completed the experiment.

Data analysis

Data were analysed in three steps using the statistical software SPSS 23.0. First, the differences in response rates, response latencies and risk rating scores of the two driver groups were analysed. Response rate was calculated as the rate of the correctly responded hazards in the video clips to the number of total hazards in the test [11]. Response latency was calculated as the time from hazard onset to the moment that participants reacted to the hazard [3–5]. Missing data in response latency in a specific video clip were replaced with the mean response time plus 3 times the standard deviation of participants who have responded [21]. Second, correlations between drivers' age, driving experience, response rate, response latency, risk rating score and confidence in answers under each hazard type were analysed. Third, linear regressions were conducted to examine whether drivers' risk rating scores of overt or/and covert hazards corresponded to their response latencies.

Results

Response rate

Response rates across hazard type and driver groups are shown in Fig 1.

A 2×2 analysis of variance (ANOVA) revealed that the main effect of driving experience was significant, $F(1, 73) = 14.85$, $p < 0.01$, $\eta 2\, p = 0.169$. Experienced drivers made more responses to hazards than novice drivers. The main effect of hazard type was significant, $F(1, 73) = 5.35$, $p < 0.05$, $\eta 2\, p = 0.068$. Drivers made more responses to overt hazards than covert hazards. The interaction between driving experience and hazard type was not significant, $F(1, 73) = 0.93$, $p > 0.05$.

Response latency

Response latencies across hazard type and driver groups are shown in Fig 2.

A 2×2 analysis of variance (ANOVA) revealed that the main effect of driving experience was significant, $F(1, 73) = 33.68$, $p < 0.01$, $\eta 2\, p = 0.316$. Experienced drivers responded to hazards faster than novice drivers. The main effect of hazard type was significant, $F(1, 73) = 57.33$, $p < 0.01$, $\eta 2\, p = 0.440$. Drivers responded to overt hazards faster than covert hazards. The interaction between driving experience and hazard type was significant, $F(1, 73) = 7.93$, $p < 0.01$, $\eta 2\, p = 0.098$. For both overt and covert hazards, novice drivers responded more slowly than experienced drivers, $F(1, 73) = 35.27$, $p < 0.01$, $\eta 2\, p = 0.326$, $F(1, 73) = 20.06$, $p < 0.01$, $\eta 2\, p = 0.216$. The effect size of driving experience on overt hazards was slightly larger than that on covert hazards.

Score of risk rating

Risk rating scores across hazard type and driver groups are shown in Fig 3.

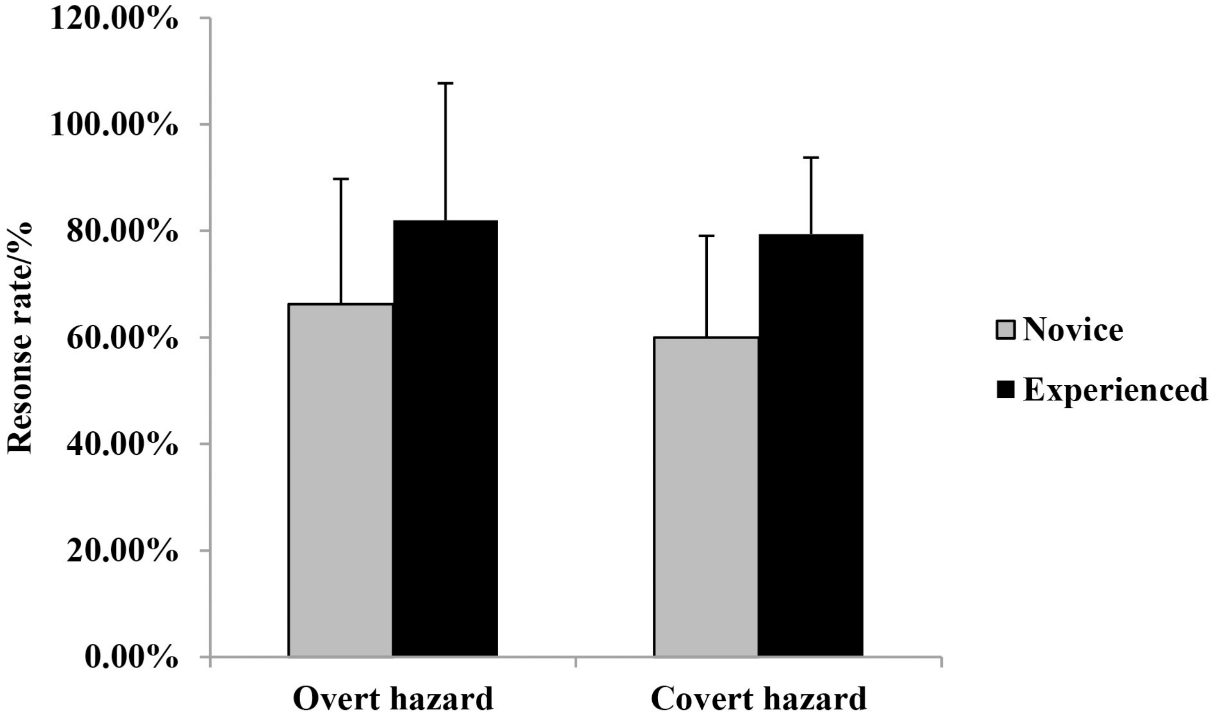

Fig 1. Response rates(means and standard errors) across hazard type and driver groups.

https://doi.org/10.1371/journal.pone.0214226.g001

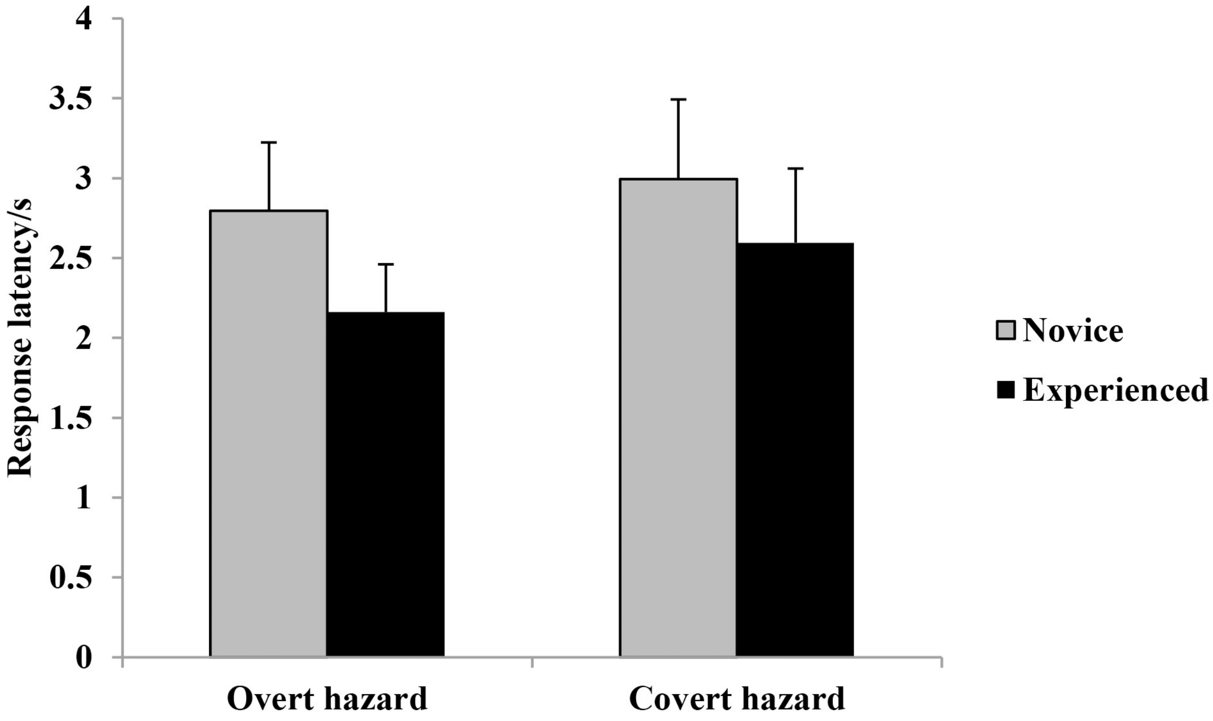

Fig 2. Response latencies(means and standard errors) across hazard type and driver groups.

https://doi.org/10.1371/journal.pone.0214226.g002

A 2×2 analysis of variance (ANOVA) revealed that the main effect of driving experience was significant, F (1, 73) = 21.47, $p < 0.01$, $\eta 2\,p = 0.227$. Experienced drivers' risk rating scores were higher than those of novice drivers. The main effect of hazard type was significant, F (1, 73) = 6.92, $p < 0.01$, $\eta 2\,p = 0.087$. Drivers' risk rating scores for covert hazards were higher than those for overt hazards. The interaction between driving experience and hazard type was not significant, F (1, 73) = 0.17, $p > 0.05$.

Correlation analysis

The correlations between drivers' age, driving experience, response rates, response latencies, risk rating scores and confidence in answers are shown in Table 2 and Table 3 for overt hazards and covert hazards, respectively.

Table 2 and Table 3 show that age and driving experience were positively correlated with response rate, risk rating and confidence in answers and negatively correlated with response latency regardless of hazard type. These results indicated that drivers' hazard perception ability improved with increasing driving experience and age. Risk rating was positively correlated with response rate regardless of hazard type, indicating that drivers with higher risk ratings for the hazards present in the video clips responded more to the hazards.

Drivers' risk rating scores were negatively and significantly correlated with their response latencies to covert hazards. Although a negative correlation was also found between risk rating scores and response latency to overt hazards, the coefficient was not significant. When the within-group correlations were calculated separately for each driver group, risk rating scores were negatively correlated with response latencies for experienced drivers (r = -0.04, $p > 0.05$, r = -0.36, $p < 0.05$, for overt hazards and covert hazards, respectively) and positively with response latencies for novice drivers(r = 0.21, $p > 0.05$, r = 0.10, $p > 0.05$). The results indicated

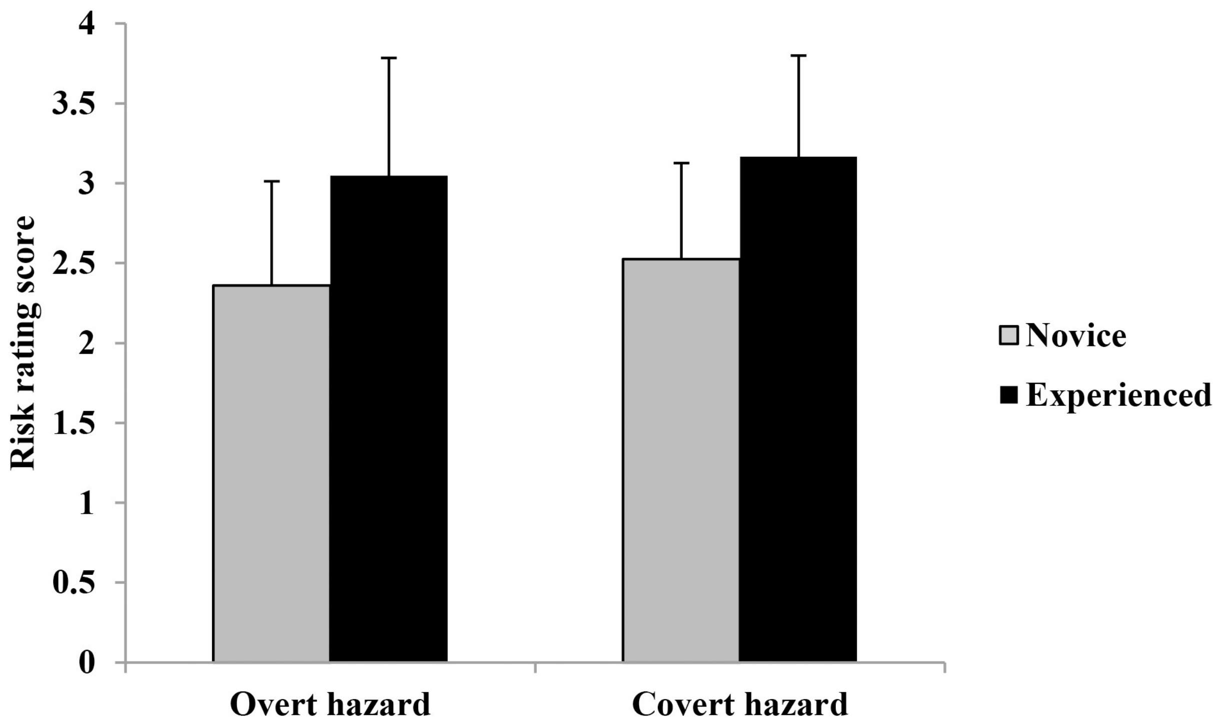

Fig 3. Risk rating scores(means and standard errors) across hazard type and driver groups.

https://doi.org/10.1371/journal.pone.0214226.g003

that the accuracies of risk ratings varied within driver groups. Confidence in answers for risk ratings were negatively correlated only with response latency to overt hazards. No significant correlations were found between drivers' risk ratings and their confidence in answers regardless of hazard type, suggesting that some drivers did not rate the risk level of hazards accurately. Additionally, there were no significant gender differences in response rate, response latency and risk rating.

To further reveal the predictors of response latency, linear regressions (Method: Stepwise) were conducted with response latency as dependent variable, risk rating and demographic factors as independent variables. Results show that driving experience and risk ratings of covert hazards can significantly predict drivers' response latency and explain 17.8% and 3.2% of the variance respectively (β = -0.38, $p<$ 0.01, β = -0.21, $p<$ 0.05). Driving experience can significantly predict drivers' response latency to overt hazards and explain 19.7% of the variance (β =

Table 2. Correlations between variables for overt hazards (n = 75).

Variables	Driving experience	Age	Response rate	Response latency	Risk rating
Age	0.88**				
Response rate	0.34**	0.31**			
Response latency	-0.46**	-0.41**	-0.40**		
Risk rating	0.35**	0.43**	0.27*	-0.21	
Confidence in answers	0.28*	0.38**	-0.08	-0.24*	0.02

Note
*$p<0.05$
**$p<0.01$

https://doi.org/10.1371/journal.pone.0214226.t002

Table 3. Correlations between variables for covert hazards (n = 75).

Variables	Driving experience	Age	Response rate	Response latency	Risk rating
Age	0.88**				
Response rate	0.32**	0.34**			
Response latency	-0.44**	-0.31**	-0.38**		
Risk rating	0.25*	0.28*	0.26*	-0.31**	
Confidence in answers	0.27*	0.37**	-0.05	-0.14	0.03

Note
*p<0.05
**p<0.01

https://doi.org/10.1371/journal.pone.0214226.t003

-0.46, $p < 0.01$). Drivers' risk ratings of overt hazards cannot predict their response latency (β = -0.06, $p > 0.05$). Similarly, driving experience can significantly predict drivers' average response latency under the two hazard types and explain 21.8% of the variance (β = -0.48, $p < 0.01$). Drivers' average risk ratings was not a good predictor (β = -0.16, $p > 0.05$).

Discussion

To support the notion that hazard types in the video clips partially account for the inconsistency in drivers' risk ratings and their response latencies in the literature, the present study 1) examines the differences in risk ratings and response latencies between novice drivers and experienced drivers; 2) reveals the differences in drivers' risk ratings and response latencies to overt and covert hazards; and 3) explores the relationships between drivers' risk ratings and their response latencies under the two hazard types.

First, the present study showed significant experience-related differences in response rates and response latencies, with experienced drivers responded more to hazards and responded to hazards faster than novice drivers. These findings were observed mainly because drivers' situation awareness developed with increasing driving experience. Hence, more experienced drivers have a better ability to anticipate other road users' trajectories or monitor the critical areas in which a potential hazard might appear or be hidden in different driving situations [22,23].

In line with some earlier studies [9,16], this study also found that experienced drivers rated the risk level of the hazards higher than novice drivers. One explanation for this result was that drivers in the present study had enough time to observe the hazards in each video clip and then rated the risk level of the hazards [16]. In contrast, experienced drivers and novice drivers were not significantly different in their risk rating scores under time-constraining conditions, such as blacking out of the video for a very short time [24]. In addition, the associations between drivers' risk rating scores and their confidence in those answers were not significant, indicating that some drivers in the present study were overconfident about their driving abilities [6, 15]. This over confidence may have in turn resulted in a lower risk rating score, regardless of hazard type.

Second, the present study showed the differences in response rates and response latencies were significant under the two hazard types. Drivers made fewer responses to covert hazards than to overt hazards, and they responded to covert hazards more slowly. Due to the nature of overt hazards, they were relatively easier for drivers to detect and respond to [18,20]. Moreover, experienced drivers responded to both overt hazards and covert hazards faster than did novice drivers. The results suggested that the influencing mechanism of driving experience on overt and covert hazards was similar, but the effect size of driving experience on overt hazards was slightly larger than that on covert hazards.

Importantly, drivers rated the risk level of overt hazards lower than that of covert hazards. This was an indication that hazard type did influence drivers' risk ratings and it might serve as an important factor in explaining the inconsistency in risk ratings in previous studies. Another indication in this study was that age and driving experience correlated positively with drivers' risk ratings regardless of hazard type. Notably, the coefficients were moderate for overt hazards, while those for covert hazards were small. There are, however, contradicting results showing that the correlations between drivers' driving experience and their risk rating scores were not significant [15].

Third, the present study demonstrated that the correlations between drivers' risk ratings and response latencies varied with hazard types. A negative and significant correlation was found between drivers' risk rating of covert hazards and their response latency, but the same result was not found for overt hazards. The result found on overt hazards was supported by two earlier studies showing no significant correlations between drivers' risk ratings and their prediction accuracies of hazards in the video clips [15,24]. In those studies, participants were asked to predict what the hazardous event was after the video clips paused or were cut to black at a certain point [15,24], which posed more time pressure on participants than did the present study.

Furthermore, drivers' higher risk ratings of covert hazards resulted in faster responses in the present study, which cannot be found on overt hazards. When considering both overt and covert hazards, drivers' average risk ratings did not correspond to their average response latencies in the task. One explanation for these results, as the data suggested, was that the accuracies of risk ratings varied within driver groups. The present study found positive correlations between risk ratings and response latencies for novice drivers regardless of hazard type, though the coefficients were not significant. These results suggest that novice drivers either cannot rate the risk level of hazards accurately or cannot translate the information about the risk of hazards into actions faster. Surprisingly, experienced drivers' risk ratings were negatively correlated with their response latencies, though the coefficient was not significant on overt hazards. For the first time, the present study found that even experienced drivers in China cannot rate the risk level of overt hazards accurately. Given these findings, video-based hazard perception trainings including measures to improve the accuracy of risk rating of different hazards might be more effective for Chinese drivers.

The results regarding the prediction of drivers' risk ratings on response latencies could be also partially explained by the crash likelihood and severity of the outcome of the hazards [16]. Compared to overt hazards, drivers in the present study may not have enough time to detect and monitor the materialization of covert hazards and fully appraise the crash likelihood and severity of the outcome [16]. This may increase the risk level of covert hazards that they experienced. For overt hazards, the result of regression showed that drivers' response latencies mainly associate with driving experience not risk ratings. Given the nature of the hazards in the present study, it is reasonable to assume that drivers' response latencies to the overt hazards may be more likely to associate with visual detection, while response latencies to the covert hazards are more likely to associate with both visual detection and risk ratings.

One limitation of this study should be acknowledged. Due to the fact that hazards in the video clips were natural ones and filmed in real driving, it is difficult to guarantee overt hazards and covert hazards were equally away from the camera car when first appeared. However, as in previous studies [4,13], the present study found that the mean length of the hazard window for each hazard type was not significant. This result indicated that driver's distance to the hazards for each hazard type was not significant given that the speed of the camera car was similar across the video clips. Further studies, probably conducted in a valid driving simulator,

are recommended to explore the effect of driver's distance to the hazards on their risk ratings and hazard response.

Conclusions

The present study provided more evidence for the notion that the inconsistency in risk ratings and response latencies in previous studies can partially attribute to the hazard type in the video clips. While experienced drivers out performed novice drivers in risk ratings and response latencies, the effects of driving experience varied by hazard type. The present study was also the first to demonstrate that hazard types underpinned the associations between drivers' risk ratings and their response latencies. Drivers' higher risk ratings of the covert hazards facilitated their response latencies, while risk ratings of the overt hazards did not correspond to their response latencies. Hazard perception trainings are needed to improve the accuracies of drivers' risk ratings of different road hazards and the coordination between their risk ratings and response latencies.

Acknowledgments

The authors would like to thank Professor Chang Ruosong for his support of this study.

Author Contributions

Data curation: Lingsen Hua.

Writing – original draft: Long Sun.

References

1. Wetton MA, Horswill MS, Hatherly C, Wood JM, Pachana NA, Anstey KJ. The development and validation of two complementary measures of drivers' hazard perception ability. Accid Anal Prev. 2010; 42: 1232–1239. https://doi.org/10.1016/j.aap.2010.01.017 PMID: 20441837
2. Crundall D. Hazard prediction discriminates between novice and experienced drivers. Accid Anal Prev. 2016; 86: 47–58. https://doi.org/10.1016/j.aap.2015.10.006 PMID: 26513336
3. Yeung JS, Wong YD. Effects of driver age and experience in abrupt-onset hazards. Accid Anal Prev. 2015; 78: 110–117. https://doi.org/10.1016/j.aap.2015.02.024 PMID: 25756847
4. Lim PC, Sheppard E, Crundall D. Cross-cultural effects on drivers' hazard perception. Transp Res F Traffic Psychol Behav. 2013; 21: 194–206. https://doi.org/10.1016/j.trf.2013.09.016
5. Huestegge L, Skottke E, Anders S, Müsseler J, Debus G. The development of hazard perception: Dissociation of visual orientation and hazard processing. Transp Res F Traffic Psychol Behav. 2010; 13: 1–8.https://doi.org/10.1016/j.trf.2009.09.005
6. Sun L, Chang R. Effects of self-assessed ability and driving experience on hazard perception. J Psychol Sci. 2016; 39: 1346–1352. https://doi.org/10.16719/j.cnki.1671-6981.20160610
7. Ba Y, Zhang W, Chan AHS, Zhang T, Cheng ASK. How drivers fail to avoid crashes: A risk-homeostasis/perception-response (RH/PR) framework evidenced by visual perception, electrodermal activity and behavioral responses. Transp Res F Traffic Psychol Behav. 2016; 43: 24–35.http://dx.doi.org/10.1016/j.trf.2016.09.025
8. Kübler TC, Kasneci E, Rosenstiel W, Schiefer U, Nagel K, Papageorgiou E. Stress-indicators and exploratory gaze for the analysis of hazard perception in patients with visual field loss. Transp Res F Traffic Psychol Behav. 2014; 24: 231–243.https://doi.org/10.1016/j.trf.2014.04.016
9. Scialfa CT, Borkenhagen D, Lyon J, Deschênes M, Horswill M, Wetton M. The effects of driving experience on responses to a static hazard perception test. Accid Anal Prev. 2012; 45: 547–553. https://doi.org/10.1016/j.aap.2011.09.005 PMID: 22269541
10. Wallis TS, Horswill MS. Using fuzzy signal detection theory to determine why experienced and trained drivers respond faster than novices in a hazard perception test. Accid Anal Prev. 2007; 39: 1177–1185. https://doi.org/10.1016/j.aap.2007.03.003 PMID: 17920841
11. Sagberg F, Bjørnskau T. Hazard perception and driving experience among novice drivers. Accid Anal Prev. 2006; 38: 407–414. https://doi.org/10.1016/j.aap.2005.10.014 PMID: 16313881

12. Crundall D, Chapman P, Phelps N, Underwood G. Eye movements and hazard perception in police pursuit and emergency response driving. J Exp Psychol. 2003; 9: 163–74.https://doi.org/10.1037/1076-898X.9.3.163

13. Chapman PR, Underwood G. Visual search of driving situations: Danger and experience. i-Perception. 1998; 27: 951–964. http://dx.doi.org/10.1068/p270951

14. Egea-Caparrós DA, García-Sevilla J, Pedraja MJ, Romero-Medina A, Marco-Cramer M, Pineda-Egea L. Late detection of hazards in traffic: A matter of response bias? Accid Anal Prev. 2016; 94: 188–197. https://doi.org/10.1016/j.aap.2016.06.002 PMID: 27328018

15. Lim PC, Sheppard E, Crundall D. A predictive hazard perception paradigm differentiates driving experience cross-culturally. Transp Res F Traffic Psychol Behav. 2014; 26: 210–217.https://doi.org/10.1016/j.trf.2014.07.010

16. Borowsky A, Oron-Gilad T. Exploring the effects of driving experience on hazard awareness and risk perception via real-time hazard identification, hazard classification, and rating tasks. Accid Anal Prev. 2013; 59: 548–565. https://doi.org/10.1016/j.aap.2013.07.008 PMID: 23954689

17. Ābele L, Haustein S, Møller M, Martinussen LM. Consistency between subjectively and objectively measured hazard perception skills among young male drivers. Accid Anal Prev. 2018; 118: 214–220. https://doi.org/10.1016/j.aap.2018.02.022 PMID: 29502852

18. Crundall D, Chapman P, Trawley S, Collins L, Van Loon E, Andrews B, et al. Some hazards are more attractive than others: Drivers of varying experience respond differently to different types of hazard. Accid Anal Prev. 2012; 45: 600–609. https://doi.org/10.1016/j.aap.2011.09.049 PMID: 22269547

19. Sun L, Li S, Chang R. Development and validity of Chinese hazard perception test. Lecture Notes Electr Eng. 2019; 527: 257–262. https://doi.org/10.1007/978-981-13-2481-9_30

20. Vlakveld WP. A comparative study of two desktop hazard perception tasks suitable for mass testing in which scores are not based on response latencies. Transp Res F Traffic Psychol Behav. 2014; 22: 218–231. https://doi.org/10.1016/j.trf.2013.12.013

21. Horswill MS, Helman S. A behavioral comparison between motorcyclists and a matched group of non-motorcycling car drivers: factors influencing accident risk. Accid Anal Prev. 2003; 35: 589–597. https://doi.org/10.1016/S0001-4575(02)00039-8 PMID: 12729822

22. Underwood G, Ngai A, Underwood J. Driving experience and situation awareness in hazard detection. Saf Sci. 2013; 56: 29–35.https://doi.org/10.1016/j.ssci.2012.05.025

23. Borowsky A, Shinar D, Oron-Gilad T. Age, skill, and hazard perception in driving. Accid Anal Prev. 2010; 42: 1240–1249. https://doi.org/10.1016/j.aap.2010.02.001 PMID: 20441838

24. Jackson L, Chapman P, Crundall D. What happens next? Predicting other road users' behaviour as a function of driving experience and processing time. Ergo. 2009; 52: 154–164.https://doi.org/10.1080/00140130802030714
